# Optimization of Sutab Preparation for Colonoscopy in Gastric Ulcer Cases: A Case Report

**DOI:** 10.7759/cureus.107668

**Published:** 2026-04-24

**Authors:** Riddhi Machchhar, Saif AlShobaki, Ahmed D Al Mahrizi, Fatima Mossolem, Alexandra Greenberg

**Affiliations:** 1 Internal Medicine, Ocean University Medical Center, Brick, USA; 2 Internal Medicine, Hackensack Meridian Ocean Medical Center, Brick, USA; 3 Education, Futures Forward Research Institute, Toms River, USA; 4 Faculty of Medicine and Surgery, University of Malta, Msida, MLT; 5 Graduate Medical Education, Praxis Institute, Voorhees, USA; 6 Gastroenterology, Hackensack Meridian Ocean Medical Center, Brick, USA

**Keywords:** colonoscopy complications, gastroenterology and hepatology, internal medicine, neuropathic ulcer, sodium phosphate

## Abstract

Sutab, a sodium sulfate oral tablet, was developed as an alternative to traditional colonoscopy preparation (prep) solutions, which usually involve the ingestion of about three liters of a polyethylene glycol (PEG) solution to clear the bowels. This traditional method can be unpleasant, leading to barriers for patients seeking colonoscopies due to the large volume of distasteful fluid and resulting diarrhea. Sutab consists of 24 sodium sulfate tablets; patients are instructed to take 12 tablets the night before the procedure with at least 16 ounces of water, followed by another 12 tablets 5-6 hours prior to the colonoscopy. Patients must also consume an additional 32 ounces of water for hydration during the prep. However, as Sutab is relatively new, ongoing research is needed. A few studies indicated potential side effects, including incidences of erosive gastritis and gastric ulcers in patients who used Sutab, raising concerns about its safety, particularly for populations with existing health issues. Here we present a case of a 53-year-old woman who used this method for colonoscopy prep and experienced complications, and aim to provide suggestions on how to mitigate these side effects.

## Introduction

Colorectal cancer screening via colonoscopy is a critical preventive measure that necessitates thorough bowel preparation (prep) for optimal visualization and detection [1]. Traditionally, this preparation involves the ingestion of polyethylene glycol (PEG)-based solutions, which can be unpleasant and difficult to tolerate for patients, often leading to lower compliance rates [1,2]. Sutab, a sodium-sulfate oral tablet, has been developed as a novel alternative to conventional bowel prep methods to address these challenges [1-4]. Unlike traditional solutions that require significant volumes of liquid intake, Sutab comprises 24 tablets taken in conjunction with adequate hydration, offering a more convenient option for patients [1-4]. Initial studies have demonstrated that Sutab provides effective bowel cleansing while improving patient experience and satisfaction [1]. However, this case report highlights the need for further observation of its side effects in a larger population, as our patient experienced corrosive gastritis following the use of Sutab [1,3,4]. This underscores the importance of evaluating the safety profile of Sutab in diverse patient groups to ensure its suitability as a widespread bowel preparation alternative [2-4].

This tablet form of colonoscopy prep was compared to the traditional United States Food and Drug Administration (US FDA)-approved bowel prep solution containing polyethylene glycol (PEG3350) mixed with (PEG-EA) or other supplemental agents for bowel prep efficacy [1,2]. Bowel prep involves clearing the colonic passage for adequate visualization of the colon and its mucosa. Traditionally, roughly three liters of fluid with osmotic activity are ingested to flood the colon, leading to diarrhea [1]. As a result of this process, the solution contains electrolytes to prevent adverse events secondary to the cleanse [1,2]. Overall, patients mention drinking copious amounts of distasteful fluid with subsequent diarrhea as a barrier to completing the prep or pursuing a colonoscopy [1,2]. Thus, other methods of prep were pursued, including a hypertonic solution, which essentially risked dehydration [1], or a tablet version with a sodium phosphate base, which required ingesting an excessive amount of tablets (32-40 tablets) and was associated with rare cases of nephrocalcinosis [1]. This article was previously posted to the Authorea preprint server in May 2025.

## Case presentation

**Figure 1:**
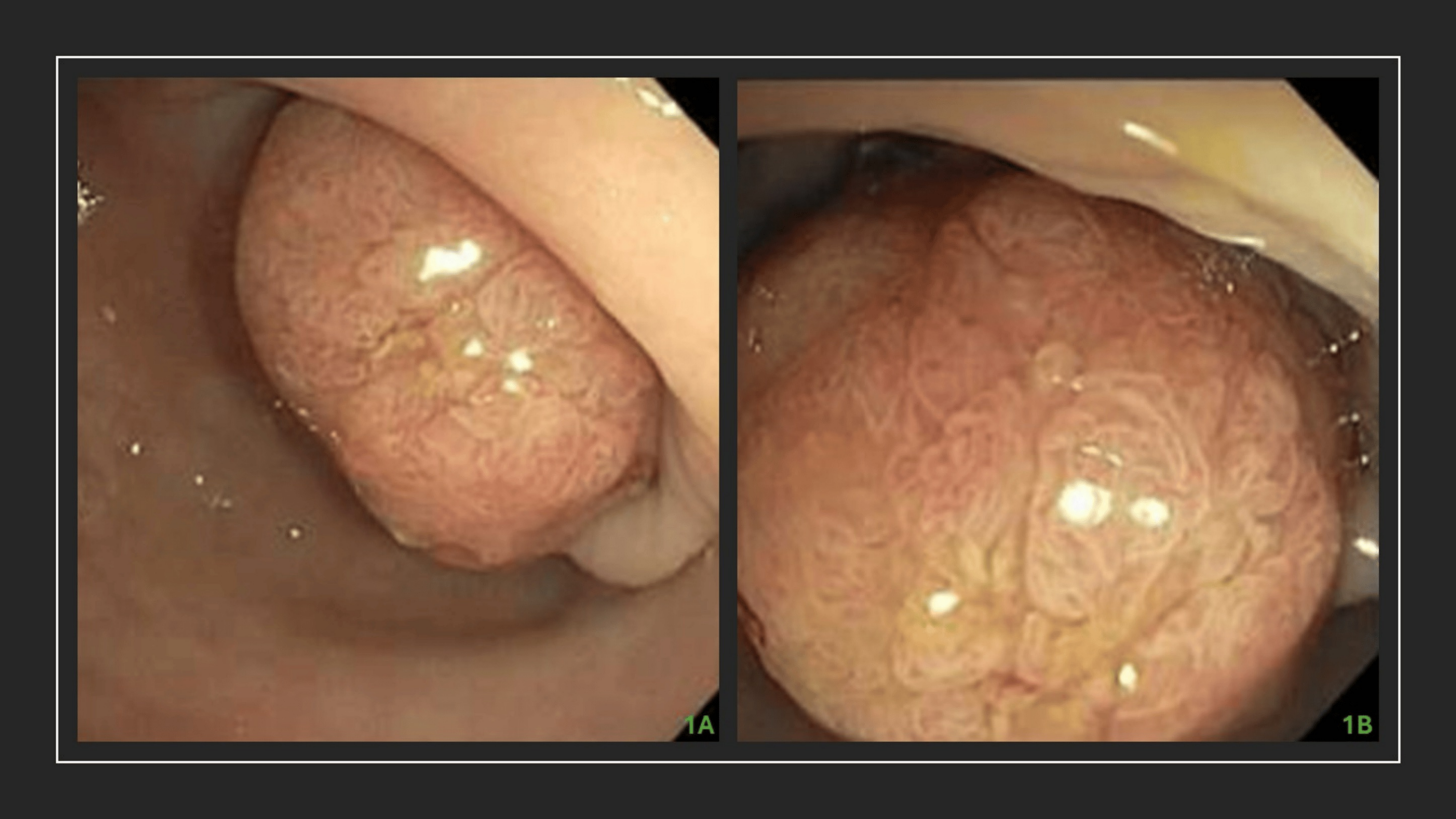
Colonoscopy images The images show a 30 mm polyp in the sigmoid colon, removed with a hot snare and retrieved. Pathology revealed a tubulovillous adenoma.

A 53-year-old female with a medical history significant for cerebrovascular accident (CVA), vascular dementia, hypertension, hyperlipidemia, prediabetes, depression, anxiety, and a childhood acute lymphoblastic leukemia (ALL) presented for a follow-up esophagogastroduodenoscopy (EGD). Three months ago, she underwent a colonoscopy for a positive Cologuard test and an abnormal CT abdomen. Her colonoscopy revealed a polyp in the sigmoid colon, with pathology revealing a tubulovillous adenoma, marked for interval assessment (Figure 1). 

**Figure 2:**
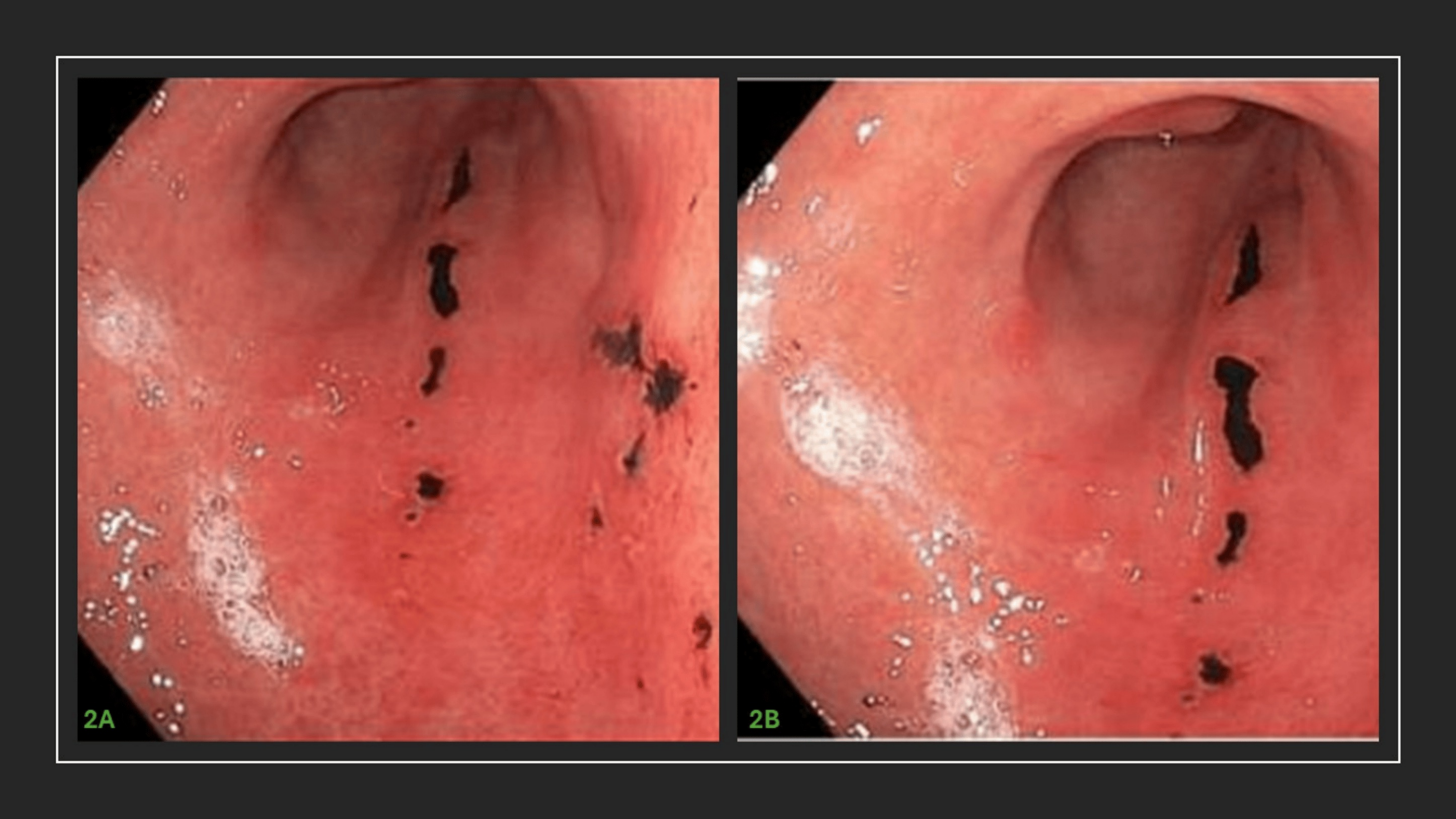
First endoscopy images First endoscopy images show antral non-bleeding gastric ulcers with pigmentation. Tissue samples were taken for biopsy.

At that time, the patient opted for Sutab pills for her colonoscopy prep instead of the traditional PEG-cleansing route. An EGD was also done due to troubling symptoms of chest and epigastric burning and discomfort. The first EGD revealed the presence of ulcerative eschars (Figure 2).

**Figure 3:**
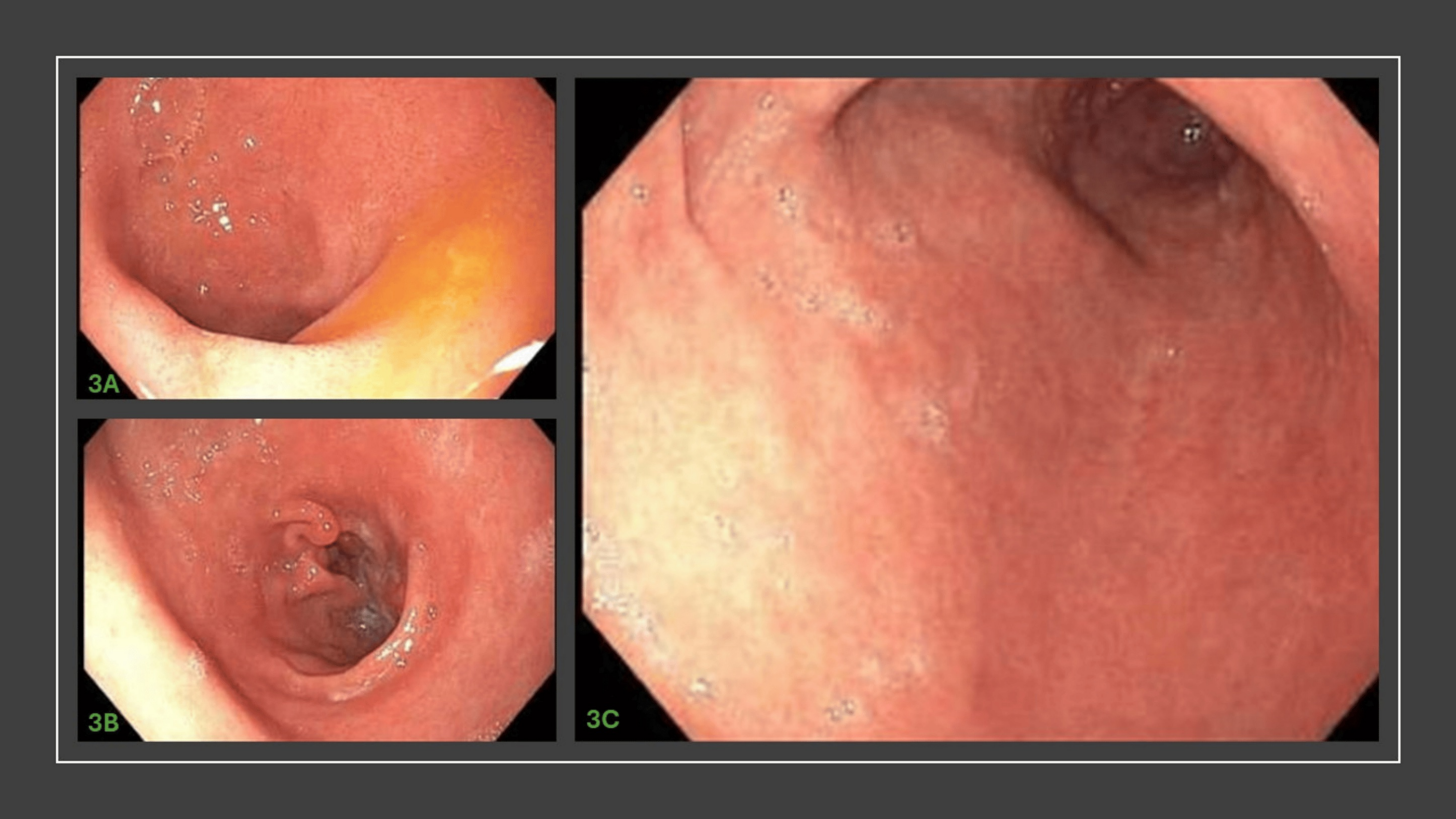
Follow-up endoscopy images. (3a-3b) Follow-up endoscopy images showing resolution of ulcers.

The patient has no history of previous ulcers, dyspepsia, or GERD until taking the oral pill prep. An EGD was subsequently performed on the same day as the colonoscopy because the patient complained of new-onset stomach pain. The clinical team was initially at a loss to explain the ulcers until discovering that they may be part of the side-effect profile of Sutab. This current EGD was performed to assess the resolution of these ulcers after the patient was treated with a twice-daily regimen of oral omeprazole 40 mg for the past three months due to persistent symptoms and patient misunderstanding. On re-evaluation with an EGD performed three months later, her eschars were resolved. In addition, after resolution of epigastric symptoms, she was able to stop her proton pump inhibitor (Figure 3).

## Discussion

FDA approval of Sutab was achieved after two randomized, investigator-blinded, active-controlled trials [2]. As mentioned, Sutab is a colon prep consisting of two dozen tablets [1,2]. A 2018 trial was done across 22 study sites with stringent inclusion and exclusion criteria. Once enrolled, these participants were randomized to receive Sutab or traditional bowel prep, and subjects maintained a treatment diary in addition to completing a survey [1]. In this trial, compliance was also checked by reviewing the amount of leftover sachets for the traditional PEG-EA prep or tablets for the Sutab prep [1]. Patient motivations for choosing Sutab were also evaluated using these data.

Gastroenterologists performing the colonoscopy were blinded to which prep their patient received and utilized the new “US FDA Bowel Prep Scoring Scale” to grade the quality of bowel cleansing in each portion of the colon from proximal to distal [1]. The 4-point scale was applied at each colon segment and was defined as such: Excellent-minimal fecal matter or fluid that was easily suctioned and overall transparent appreciation of the colon; Good-fecal and fluid matter requiring suction, but clear visualization of the mucosa is achieved; Fair-fecal matter remaining after suction, obscuring the colonic mucosa, and Poor-copious fecal matter requiring further bowel prep [1].

Of those patients allotted to the Sutab prep group, 16.2% did not complete the trial for the following reasons: Adverse event (2%), Lost to follow-up (37.3%), Physician decision (3.9%), Subject withdrew consent (27.5%), and other reasons (29.4%) [1]. 92.4% of the Sutab subjects had a successful prep compared to the 89.3% using the PEG-EA prep. The US FDA Bowel Scoring Scale for the utab patients overall showed Excellent outcomes (66.2%) with the breakdown of prep results as such: significant Excellent proximal colon (64.7%, p-value 0.034), 70.3% Excellent mid-colon, and 66.5% Excellent distal colon [1]. Analysis of noninferiority (using the 10% margin) established that the sodium-sulfate tablet was noninferior to the traditional prep (<0.001) [1]. Age, gender, and race did not contribute to overall bowel prep success. 

Regarding adverse events or symptoms related to the tablets, 77 of 281 subjects reported none, while 14 had gastrointestinal side effects evenly divided amongst mild abdominal pain, constipation, diarrhea, flatulence, and vomiting [1]. None had proctitis [1]. Of those with abdominal pain, the severity of symptoms was mild (11%) or moderate (6%), abdominal distension was either mild (21%) or moderate (9%), nausea carried from mild (35%), moderate (13%) or severe (1%), and the severity of vomiting was either mild (11%) or moderate (12%) [1]. The majority of participants on Sutab had abnormalities involving electrolytes (42 subjects) [1]. While 0.7% of these patients had renal complications, sodium-sulfate tablets are less likely to cause nephropathies as compared to a sodium-phosphate tablet; more data is necessary regarding their use in populations with heart failure, renal insufficiency, and electrolyte derangements [1,2]. Overall, on the patient preference questionnaire, subjects in the Sutab group compared to the traditional prep significantly reported that adhering to and tolerating the prep was “very easy” and “easy” (65.1%, p-value <0.001). The overall experience was “excellent” and “good” (71.6%, p-value 0.004), the process was noted to be “better” than their previous experience (65.1%, p-value <0.001), and these patients would request the prep again (78.1%, p-value 0.005). It should be noted that, unlike our patient, no one in this trial reported the development of ulcers. This could be due to the rarity of getting dual-scoped (particularly on the same day), as that is typically done only when patients have aggressive symptoms of dyspepsia or an unknown bleed [5]. Xie et al., however, noted that nausea and vomiting were more common in those taking the oral prep therapy than the traditional PEG, and noted this relationship to be statistically significant [6].

As Sutab is relatively new, significant data is still being collected on its use in the mainstream population. Two previous abstracts in 2022 describing a total of seven cases have been published regarding possible side effects of Sutab with incidental findings on endoscopies for symptoms related to its use [3,4]. The studies highlight the entire contents of Sutab (1.479 g sodium sulfate, 0.225 g magnesium sulfate, 0.188 g potassium chloride), which can lead to undocumented side effects in their trial, which granted FDA approval [2,3]. Five patients over two months in 2021 experienced a colonoscopy followed by an endoscopy for new-onset dyspepsia and epigastric discomfort with one common factor, use of Sutab as bowel prep [3]. In those EGDs, all patients had moderate to severe erosive gastritis, with two cases exhibiting linear gastric ulcers with black eschar [3]. All five patients were discharged on antacids and returned for follow-up endoscopy and evaluation of resolution of signs and symptoms, similar to our patient [3]. The second publication evaluating similar findings of frank gastritis and corrosive ulcerations in two patients on Sutab experiencing new-onset dyspepsia suggested staggering the consumption of each 12-tablet dose over time with significant hydration to potentially avoid gastritis and ulcerations [4]. This case report adds to a growing number of reports on Sutab causing linear ulcerations with eschar formation in patients undergoing colonoscopy prep with this newer medication.

## Conclusions

Sutab is a fairly novel drug that is still being studied for its use as preparation for colonoscopies, especially in comparison to the more traditional PEG therapy. Due to its novelty, its full side-effect profile is still unknown. We urge clinicians to closely monitor patients who have opted to use Sutab for any expected or unexpected adverse events. The development of an ulcer in our patient, while rare, was not a completely isolated event. As more patients opt for Sutab, it would be beneficial for clinicians to continue to publish any unexpected or rare findings so that the determination of what the most susceptible population is for these adverse events can be made.
